# Newborn screening for congenital hypothyroidism in France: a study of current professional practices

**DOI:** 10.1530/ETJ-25-0246

**Published:** 2026-03-06

**Authors:** Maelle Quenet, Stéphanie Leroux, Claude Bendavid, Marie-Béatrice Saade, Alain Beuchee, Amandine Bellanger, Caroline Moreau

**Affiliations:** ^1^ Service de Néonatalogie, Hôpital Sud CHU Rennes-France, Rennes, France; ^2^ Service de Pédiatrie; Hôpital Sud CHU Rennes University Rennes, CHU Rennes, Inserm, EHESP, Irset – UMR_S 1085, Rennes, France; ^3^ Service de Biochimie-Toxicologie, Hôpital Pontchaillou CHU Rennes-France – Numecan INSERM Rennes, Rennes, France; ^4^ Service d’Endocrinologie Pédiatrique, Hôpital Sud CHU Pontchaillou Rennes-France, Rennes, France; ^5^ Service de Néonatalogie, Hôpital Sud CHU Pontchaillou Rennes-France, Rennes, France; ^6^ Service de Biochimie-Toxicologie; Hôpital Pontchaillou CHU Rennes – University Rennes, CHU Rennes, INSERM, EHESP, IRSET (Institut de Recherche en Santé, Environnement et Travail) UMR_S 1085, Rennes, France

**Keywords:** congenital hypothyroidism, neonatal screening, thresholds, algorithms, preterm

## Abstract

**Objective:**

Congenital hypothyroidism is a leading cause of preventable intellectual disability. Neonatal screening enables early detection and treatment, ideally before symptoms appear. In France, screening has been part of national public health policy since 1978, relying on standardized protocols to ensure equity of care. However, the actual adherence to these guidelines and potential local adaptations remain undocumented. This study aimed to describe current practices of French Regional Neonatal Screening Centers and assess their conformity with national recommendations.

**Methods:**

In 2024, a cross-sectional descriptive survey was conducted via a structured online questionnaire sent to all 16 French Regional Neonatal Screening Centers. The questionnaire addressed TSH retesting and referral thresholds, use of thyroxine assays, second-tier testing, resampling protocols, and management of preterm infants. Data were analyzed to identify deviations from guidelines and their potential implications.

**Results:**

All 16 centers responded (100% response rate). Nine (56%) reported deviations from national recommendations. Five centers applied alternative TSH retesting or referral thresholds. Four measured thyroxine on the first dried blood spot for intermediate TSH levels, with one retrospectively evaluating this approach. Three centers had specific resampling protocols for preterm infants, but none used gestational age-adjusted TSH thresholds. These adaptations aimed to reduce false positives and false negatives associated with the standard protocol.

**Conclusion:**

This nationwide survey highlights substantial heterogeneity in congenital hypothyroidism screening practices across France. Variations in TSH thresholds, thyroxine measurement, and preterm infant management may impact diagnostic consistency and equity. Harmonization efforts based on evidence are needed to optimize neonatal screening performance nationally.

## Introduction

Congenital hypothyroidism (CH) is a dysfunction of the hypothalamic–pituitary–thyroid axis present at birth, caused either by abnormal thyroid gland development or hormone synthesis defects (primary CH) or by insufficient pituitary stimulation of a normal gland (central CH). Primary CH usually results in low circulating thyroid hormone levels and elevated thyroid-stimulating hormone (TSH) concentrations, whereas central CH may occur alone or with other pituitary hormone deficiencies. Thyroid hormones are critical for brain development, bone maturation, and postnatal growth (1).

In France, CH affects approximately 1 in 2,000–4,000 live births (2). Since its inclusion in the national newborn screening (NBS) program in 1978, over 30 million infants have been screened and about 10,000 diagnosed. Although mortality is low, untreated CH leads to irreversible neurodevelopmental impairment, including intellectual disability and motor deficits (3). Early detection and rapid initiation of hormone replacement are therefore crucial.

Worldwide, only about 30% of newborns currently benefit from CH screening (4), and protocols vary widely. Some programs measure total thyroxine (T4) with reflex TSH testing, enabling detection of both primary and central CH but with higher false-positive (FP) rates. Others – such as France – rely exclusively on TSH measurement, which detects only primary CH (5). According to French guidelines, dried blood spot samples are collected between 48 and 72 h of life and analyzed in the designated regional laboratory. Abnormal TSH is defined as ≥20 mIU/L with the Autodelfia method or ≥17 mIU/L with the GSP method (Revvity®, USA) (2), thresholds among the highest internationally, as many programs use cutoffs between 6 and 12 mIU/L (6, 7, 8). The 2021 European Society for Paediatric Endocrinology (ESPE) guidelines recommend alternative algorithms combining TSH and T4 to optimize sensitivity (9).

Preterm newborns are particularly vulnerable to transient CH and delayed TSH rise due to immaturity of the hypothalamic–pituitary–thyroid axis. This can result in false-negative (FN) results when screening is performed only once at 48 h. ESPE guidelines therefore recommend a second dried blood spot at two weeks of age for high-risk groups (preterm, low birth weight, sick newborns, and multiple births) (9). However, these recommendations are not uniformly implemented across Europe, and France has not adopted them.

Despite the existence of a national protocol, marked regional variations in CH screening persist in France. This is particularly concerning because the French NBS program is organized on a regional basis but coordinated nationally by the *Centre National de Coordination du Dépistage Néonatal* in collaboration with the National Biology Commission. As stated in the *Arrêté du 22 février 2018* (*relatif à l’organisation du programme national de dépistage néonatal recourant à des examens de biologie médicale – Légifrance*), one of their explicit missions is to ensure uniform screening across the entire country so that every newborn, regardless of birthplace, receives the same standard of care. Persistent disparities therefore represent a deviation from both the intended organizational model and the legal framework. No recent nationwide evaluation of the concordance between French neonatal screening practices and national guidelines has been conducted since the publication of this legal text.

The aim of this study was to provide a detailed overview of CH screening algorithms implemented in each French region, in order to inform national decision-making toward a more consistent, evidence-based, and equitable protocol.

## Materials and methods

### Organization of newborn screening in France

Since 2018, biological newborn screening in France has been coordinated nationally by the *Centre National de Coordination du Dépistage Néonatal* (CNCDN) in close collaboration with the National Biology Commission, with the explicit mission – mandated by the *Arrêté du 22 février 2018* (*relatif à l’organisation du programme national de dépistage néonatal recourant à des examens de biologie médicale – Légifrance*) – to ensure uniform screening practices across all regions. Each administrative region hosts a Regional Neonatal Screening Center named *Centre Régional de Dépistgae Néonatal* (CRDN) responsible for testing samples from its area; overseas territories are affiliated with mainland centers (Paris for Guadeloupe and Martinique; Lille for French Guiana, Mayotte, and Réunion). In total, 16 laboratories currently perform NBS for the entire French population.

### National screening protocol for CH

The French protocol, defined by national legislation, relies exclusively on TSH measurement in dried blood spots collected at 48–72 h of life. Thresholds depend on the immunoassay method:

- Retest threshold: 15 mIU/L (Autodelfia®) or 12 mIU/L (GSP®).
- Action threshold: 20 mIU/L (Autodelfia®) or 17 mIU/L (GSP®).

**Figure 1:**
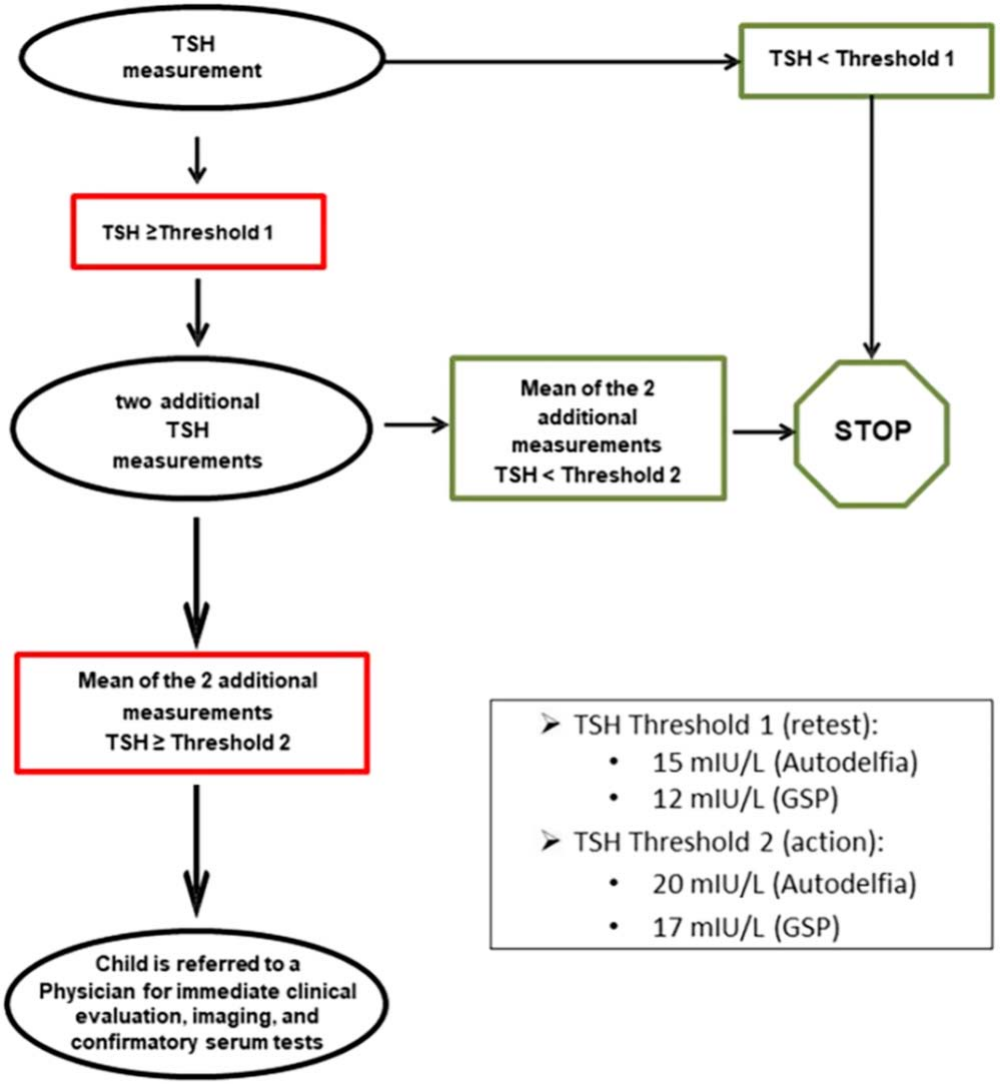
National algorithm recommended by French legislation for CH newborn screening.

If the initial TSH exceeds the retest threshold, two additional measurements are performed on the same card. If the mean of these exceeds the action threshold, the referring physician is contacted for immediate clinical evaluation, imaging, and confirmatory serum tests (Fig. 1).

### Survey design

In July 2024, we conducted a cross-sectional descriptive survey using a Google Forms® questionnaire sent to all 16 CRDN. The form included 14 items, primarily multiple-choice, with optional open-text fields for clarification. When needed, additional details were obtained by individual email follow-up. For anonymity, each center was assigned a number in all tables and figures.

### Study design and reporting guidelines

This observational descriptive study is based on a national survey of professional practices and a contextual analysis of international screening policies. No patient-level or identifiable data were used. The study followed the STROBE guidelines for observational studies and the RECORD extension for routinely collected data; the completed RECORD checklist is provided as supplementary material.

### Ethical considerations

The study was conducted in accordance with the Declaration of Helsinki (2013 revision). The Clinical Research Department of CHU Rennes reviewed the protocol and confirmed that formal ethical approval was not required. All data were anonymized and aggregated at the center level. Written agreement for the use and publication of responses was obtained from each CRDN. The CNCDN was informed of the study, and documentation of the institutional waiver is available upon request.

## Results

We obtained responses from all sixteen CRDN involved in CH screening in France, representing full national coverage (Table 1).

**Table 1:** Anonymization table for French Regional Neonatal Screening Centers, with incidence of CH. These data come from the 2023 activity report of the National Coordination Center for Neonatal Screening and is freely available on its website (*Rapports D’activités du dépistage néonatal*) (2).

Regional neonatal screening number	Number of births in 2023	Incidence of CH from the start of newborn screening in 1978	Incidence of CH in 2023
1A + 1B	78,785	1/3,150	1/2,918
2	22,859	1/2,646	1/2,078
3	29,397	1/2,692	1/2,672
4	22,239	1/3,126	1/2,022
5A + 5B	47,782	1/2,825	1/2,172
6A + 6B	58,877	1/2,832	1/2,181
7	155,958	1/3,061	1/2,437
8	30,076	1/3,240	1/3,760
9	49,843	1/3,143	1/2,266
10A + 10B	51,022	1/4,034	1/2,572
11	54,022	1/3,263	1/2,843
12	36,240	1/3,464	1/2,416

When asked whether their neonatal screening protocol differed from the one recommended by French legislation (*Arrêté du 22 février 2018 relatif à l’organisation du programme national de dépistage néonatal recourant à des examens de biologie médicale – Légifrance*), nine centers (56%) confirmed that they had implemented specific adaptations, while seven centers (44%) adhered strictly to the national guidelines.

### Variations in TSH thresholds

Five centers reported deviations from the national TSH thresholds for retesting and action. For example, Center 5B requests a second dried blood spot (DBS) if TSH is between 15 and 20 mIU/L (Autodelfia method), aiming to reduce FN. Centers 7, 8, and 9 implemented higher referral thresholds on the first DBS (ranging from 25 to 34 mIU/L, depending on the center and assay), combined with delayed second DBS collections at 8 or 15 days of life. These strategies aim primarily to reduce FP and unnecessary recalls. Center 12 lowered the retest threshold to 14 mIU/L (Autodelfia) and adjusted the referral criteria on the second DBS to increase sensitivity and reduce FN (Table 2).

**Table 2:** Summary of neonatal screening practices in France differing from national recommendations for congenital hypothyroidism.

Domain/center(s)	Adaptation/specific practice	Objective
TSH thresholds differing from recommendations		
5B	Second DBS if TSH 15–20 mIU/L (Autodelfia)	Reduce FN
7	First DBS: refer only if TSH ≥ 34 mIU/L (GSP); second DBS at day 8 if 17–34 mIU/L; notify MD if TSH ≥ 8.5 mIU/L on second DBS	Reduce FP
8	First DBS: refer only if TSH ≥ 34 mIU/L (GSP); second DBS at day 15 if 17–34 mIU/L; notify MD if TSH ≥ 8 mIU/L on second DBS	Reduce FP
9	First DBS: refer only if TSH ≥ 25 mIU/L (GSP); second DBS if 17–25 mIU/L; notify MD if TSH ≥ 8.5 mIU/L on second DBS	Reduce FP
12	Retest threshold lowered to 14 mIU/L (Autodelfia); second DBS if 14–20 mIU/L; notify MD if TSH ≥ 10 mIU/L on second DBS	Reduce FN
Use of T4 on DBS		
1A, 1B, 2	Total T4 measured if TSH between 12 and 17 mIU/L (GSP) or between 15 and 20 mIU/L (Autodelfia); refer only if total T4 ≤ 40 nmol/L	Detect FN
8	Free T4 (RIA) if TSH 12–17 mIU/L (GSP); second DBS if free T4 < 10 pmol/L	Reduce FN
7	Retrospective total T4 measurement if TSH 12–15 mIU/L (GSP)	Under evaluation
Specific protocols for preterm infants
6B	Second DBS at day 15 if GA < 33 weeks	Avoid FN
7	Second DBS at day 21	Avoid FN
12	Second DBS at corrected GA = 32 weeks if born <32 weeks	Avoid FN
TSH thresholds for preterms		
None	—	—

DBS, dried blood spot; MD, medical doctor; GA, gestational age; GSP, GSP® Neonatal hTSH kit; RIA, radioimmunoassay; D, day of life; and FP, false positive; FN, false negative.

### Use of T4 measurement on dried blood spot

Four centers (1A, 1B, 2, and 8) incorporate T4 assays on the initial DBS when TSH levels fall between retest and action thresholds, either total T4 (immunoassay, GSP® Revvity) or free T4 (radioimmunoassay (RIA), using iodine-125). This practice is intended to identify FN that might be missed when relying solely on TSH measurement. Center 7 is conducting a retrospective study on total T4 measurement but has not yet integrated it into routine screening.

### Specific protocols for preterm infants

Three centers have adopted specific protocols for preterm newborns by systematically performing a second DBS at defined postnatal or corrected ages (day 15, day 21, or at 32 weeks corrected GA), reflecting the higher risk of delayed TSH elevation and FN in this population. Notably, no center currently uses specific TSH cutoff values for preterm infants, relying instead on timing adjustments.

## Discussion

This comprehensive survey of neonatal CH screening practices across French regional centers reveals significant heterogeneity despite a national framework designed to ensure uniformity. Over half of the centers have deviated from official guidelines, highlighting challenges in applying a standardized protocol among diverse local conditions and clinical experiences. These adaptations reflect local efforts to optimize screening performance – mainly aiming to reduce FP or FN results – while remaining within the national legal framework. Understanding these discrepancies is essential for improving screening performance and ensure equitable care for all newborns. This variability contrasts with countries such as Japan, where national guidelines (10) mandate uniform procedures, including standardized TSH cutoff values, timing of sampling, and specific protocols for premature and low birth weight infants. Nagasaki *et al.* underscore the delicate balance between minimizing FP and FN results, with these thresholds grounded in clinical observations despite the low incidence of CH. The Japanese model highlights the clinical consequences of delayed diagnosis, including intellectual disability and growth failure, thus underscoring the need for timely and accurate screening. This heterogeneity may also hamper the comparability of epidemiological data across regions, complicating national monitoring and quality assurance efforts. In contrast, a centralized and uniform framework, as exemplified by Japan, facilitates consistent training, standardized laboratory practices, prompt communication of results, and timely clinical management, thereby enhancing overall screening efficacy.

In France, the reliance on localized protocols, shaped by limited clinical experience due to the rarity of CH cases, may lead to inconsistent handling of FP and FN, potentially affecting treatment initiation and outcomes. This situation suggests a need for enhanced harmonization at the national level to ensure equitable screening quality, optimize diagnostic performance, and ultimately improve neonatal health outcomes.

One of the most notable sources of variation lies in the TSH thresholds for retesting and referral, which range widely from 14 to 34 mIU/L depending on the center and assay method (GSP or Autodelfia). The French national action threshold of 17 mIU/L (GSP method) is among the highest worldwide, whereas most countries use lower cutoffs between 6 and 12 mIU/L (6, 7, 11, 12, 13). This relatively high threshold corresponds to the 99.86th percentile with a positive predictive value of approximately 30% (7), reflecting a strategy primarily aimed at limiting FP and unnecessary follow-ups. Although our study lacks direct regional data on FP and FN, international evidence illustrates the impact of threshold adjustments on screening performance. For example, the Swedish neonatal screening program (14) demonstrated that lowering the TSH cutoff from 20 to 10 mIU/L (plasma) increased the detection rate of CH cases from 1/3,374 to 1/2,222 births – a relative increase of approximately 52%. This adjustment corresponded to a substantial decrease in FN, with the incidence of undetected CH falling from 1/2,563 to 1/7,840. This improvement in sensitivity was accompanied by a rise in FP, yet the positive predicted value remained high at 76%, outperforming many other programs. These findings exemplify the inherent trade-off between sensitivity and specificity when selecting TSH screening thresholds. Consistent with this, other international studies have reported enhanced detection rates following threshold lowering, particularly for mild or atypical CH forms. For instance, in Lombardy, reducing the cutoff from 20 to 10 mIU/L (Autodelfia method) significantly increased the identification of CH cases, particularly those with ectopic thyroid tissue or dysgenesis (15). Similarly, Gunnerbeck *et al.* (14) also reported that lowering the TSH cutoff increased the detection of CH cases and significantly reduced FN. These missed cases were more frequent among premature infants, twins, low birth weight neonates, and those with congenital anomalies, many being transient forms of CH.

Neurodevelopmental studies further complicate this balance. An Australian cohort study published in 2016 reported increased academic and developmental difficulties among children with neonatal TSH values between the 99.5th and 99.9th percentiles, with a prevalence of 11.3% compared to 5% for children below the 75th percentile (16). Such findings suggest a potential benefit in lowering screening thresholds to capture subtle hypothyroidism earlier. However, contrasting evidence comes from a 2020 New Zealand study, which found no association between mildly elevated TSH (8–14 mIU/L, Autodelfia) and lower IQ, underscoring the complexity and the need for further research (17).

In addition to threshold variability, several centers adjust their protocols according to the timing of screening. This is particularly relevant for late sampling, where the maturation of the hypothalamic–pituitary–thyroid axis can influence TSH values. For example, in the United States, lower TSH cutoffs are recommended when screening is delayed beyond 30 days of life (18). Yet, optimal thresholds for late screening remain undefined, and the relevance of dried blood spot retesting versus serum testing in this context lacks robust evidence, indicating a need for concordance studies. Premature infants represent a particularly vulnerable population where screening is even more complex. Three French centers have implemented second screenings at varying postnatal ages (day 15, day 21, or corrected gestational age of 32 weeks) consistent with recent European guidelines recommending additional testing for preterms to detect both primary and central CH (9, 19, 20). Despite this, no center in our survey has defined specific TSH cutoffs for preterm neonates, highlighting a critical gap in standardization.

The role of T4 measurements in neonatal screening further illustrates practice heterogeneity. Four centers currently measure T4 (total or free) on dried blood spots when TSH values fall between retest and referral thresholds to improve diagnostic accuracy and reduce FN. This strategy aligns with international recommendations such as those from the European Society for Paediatric Endocrinology, which endorses TSH as the primary screening marker with free T4 measurement when feasible to detect central hypothyroidism (9). Practices vary globally: some U.S. states incorporate simultaneous or sequential TSH and total T4 testing, Japan has reduced FP by adding free T4 measurement, and the Netherlands complements T4/TSH screening with thyroxine-binding globulin assays (21). Our results suggest that such reflex strategies could be considered in France, but only after formal medico-economic evaluation to balance costs and benefits.

Operational constraints and ethical considerations also shape these heterogeneous practices. Regional adaptations – while often effective locally – risk creating inequities in screening quality and access. This raises important ethical and legal questions regarding adherence to national regulations versus the implementation of locally optimized protocols. Maintaining national consistency is important to guarantee equitable, high-quality screening and early treatment, which are critical to prevent irreversible neurodevelopmental damage. Moreover, minimizing FP alleviates stress on families and reduces unnecessary healthcare burden.

Importantly, this study underscores the necessity of conducting regular, systematic evaluations of neonatal screening practices and outcomes, reflecting the principles established by Léger *et al.* in their landmark works from 1990 and 2021 (22, 23). Their recent work further highlights the growing proportion of transient CH cases with an anatomically normal thyroid gland, characterized by low levothyroxine doses at 6 months as a strong predictor. These findings resonate with our own data, where several centers reported a substantial proportion of cases ultimately classified as transient, prompting discussion about treatment duration and follow-up strategies.

This study has several limitations. First, the survey relied on self-declaration of practices by regional newborn screening centers, which may introduce response bias or incomplete reporting. Second, there was no verification of the reported data against regional screening registries, limiting the accuracy assessment of the responses. Third, due to the absence of regional data on FP and FN, it was impossible to directly evaluate the impact of protocol variations on screening performance. Fourth, the study did not include qualitative investigations that could have provided deeper insights into the reasons behind local adaptations. Notably, regional screening centers emphasized the need to limit FP and FN, but this concern was based on limited and sporadic clinical observations, given the low incidence of the disease. Finally, the cross-sectional design captures practices at a single point in time and may not reflect ongoing changes or future developments in screening protocols.

In conclusion, our findings highlight several avenues that could be explored to harmonize and optimize neonatal thyroid screening in France. Potential strategies include i) assessing the relevance of gestational-age-adapted thresholds, ii) evaluating the cost-effectiveness and clinical utility of integrating T4 testing, and iii) defining national guidelines for preterm retesting. At present, data on FP and FN are not available at a regional level, and collecting such information would represent a major step forward in improving both screening performance and the management of affected children. Centralized data collection and continuous quality monitoring would be valuable to support these developments and ensure equitable, effective, and timely screening for all newborns.

## Declaration of interest

The authors declare that there is no conflict of interest that could be perceived as prejudicing the impartiality of the work reported.

## Funding

This study received no external funding. No sponsor was involved in the study design; the collection, analysis, and interpretation of data; the writing of the manuscript; or the decision to submit it for publication.

## Author contribution statement

MQ conceived the methodology, performed the formal analysis, curated the data, conducted the investigation, managed project administration, and wrote the original draft. SL contributed to validation, reviewed and edited the manuscript, and prepared visualizations. MBS contributed to validation, reviewed and edited the manuscript, and prepared visualizations. AlB contributed to validation, reviewed and edited the manuscript, and prepared visualizations. CB contributed to validation, reviewed and edited the manuscript, and prepared visualizations. AmB conceived the methodology, supervised the study, curated the data, managed project administration, and wrote the original draft. CM conceived the methodology, supervised the study, curated the data, managed project administration, and wrote the original draft. All authors have read and approved the final version of the manuscript.

## References

[1] Klosinska M, Kaczynska A & Ben-Skowronek I. Congenital hypothyroidism in preterm newborns – the challenges of diagnostics and treatment: a review. Front Endocrinol 2022 13 860862. (10.3389/fendo.2022.860862) PMC8972126 35370986

[2] Centre National de Coodination du Dépistage Néonatal. Rapport d’activité programme national du dépisatge néonatal Année 2023, 2023. [cited 2025 Apr 1]. (https://depistage-neonatal.fr/wp-content/uploads/2025/03/Rapport-Activite-2023_vf_20250310.pdf)

[3] Hulse JA. Outcome for congenital hypothyroidism. Arch Dis Child 1984 59 23–29. (10.1136/adc.59.1.23) 6198974 PMC1628400

[4] Arrigoni M, Zwaveling-Soonawala N, LaFranchi SH, et al. Newborn screening for congenital hypothyroidism: worldwide coverage 50 years after its start. Eur Thyroid J 2025 14 e240327. (10.1530/etj-24-0327) 39812367 PMC11816049

[5] LaFranchi SH. Newborn screening strategies for congenital hypothyroidism: an update. J Inher Metab Disea 2010 33 225–233. (10.1007/s10545-010-9062-1) 20195902

[6] Ford G & LaFranchi SH. Screening for congenital hypothyroidism: a worldwide view of strategies. Best Pract Res Clin Endocrinol Metab 2014 28 175–187. (10.1016/j.beem.2013.05.008) 24629860

[7] Levaillant L, Huet F, Bretones P, et al. Neonatal screening for congenital hypothyroidism: time to lower the TSH threshold in France. Arch Pediatr 2022 29 253–257. (10.1016/j.arcped.2022.02.001) 35351343

[8] Mehran L, Khalili D, Yarahmadi S, et al. Worldwide recall rate in newborn screening programs for congenital hypothyroidism. Int J Endocrinol Metab 2017 15 e55451. (10.5812/ijem.55451) 29201074 PMC5702453

[9] Léger J, Olivieri A, Donaldson M, et al. European Society for Paediatric Endocrinology consensus guidelines on screening, diagnosis, and management of congenital hypothyroidism. J Clin Endocrinol Metab 2014 99 363–384. (10.1210/jc.2013-1891) 24446653 PMC4207909

[10] Nagasaki K, Minamitani K, Nakamura A, et al. Guidelines for newborn screening of congenital hypothyroidism (2021 revision). Clin Pediatr Endocrinol 2023 32 26–51. (10.1297/cpe.2022-0063) 36761493 PMC9887297

[11] Deladoëy J, Ruel J, Giguère Y, et al. Is the incidence of congenital hypothyroidism really increasing? A 20-year retrospective population-based study in Québec. J Clin Endocrinol Metab 2011 96 2422–2429. (10.1210/jc.2011-1073) 21632812

[12] Mengreli C, Kanaka-Gantenbein C, Girginoudis P, et al. Screening for congenital hypothyroidism: the significance of threshold limit in false-negative results. J Clin Endocrinol Metab 2010 95 4283–4290. (10.1210/jc.2010-0057) 20591982

[13] Olivieri A, Corbetta C, Weber G, et al. Congenital hypothyroidism due to defects of thyroid development and mild increase of TSH at screening: data from the Italian National Registry of infants with congenital hypothyroidism. J Clin Endocrinol Metab 2013 98 1403–1408. (10.1210/jc.2012-3273) 23443814

[14] Gunnerbeck A, Lundholm C, von Döbeln U, et al. Neonatal screening for congenital hypothyroidism in Sweden 1980–2013: effects of lowering the thyroid-stimulating hormone threshold. Eur J Endocrinol 2023 188 536–546. (10.1093/ejendo/lvad064) 37306289

[15] Corbetta C, Weber G, Cortinovis F, et al. A 7-year experience with low blood TSH cutoff levels for neonatal screening reveals an unsuspected frequency of congenital hypothyroidism (CH). Clin Endocrinol 2009 71 739–745. (10.1111/j.1365-2265.2009.03568.x) 19486019

[16] Lain SJ, Bentley JP, Wiley V, et al. Association between borderline neonatal thyroid-stimulating hormone concentrations and educational and developmental outcomes: a population-based record-linkage study. Lancet Diabetes Endocrinol 2016 4 756–765. (10.1016/s2213-8587(16)30122-x) 27453174

[17] West R, Hong J, Derraik JGB, et al. Newborn screening TSH values less than 15 mIU/L are not associated with long-term hypothyroidism or cognitive impairment. J Clin Endocrinol Metab 2020 105 dgaa415. (10.1210/clinem/dgaa415) 32598474

[18] Rose SR, Wassner AJ, Wintergerst KA, et al. Congenital hypothyroidism: screening and management. Pediatrics 2023 151 e2022060419. (10.1542/peds.2022-060420) 36827521

[19] Caiulo S, Corbetta C, Di Frenna M, et al. Newborn screening for congenital hypothyroidism: the benefit of using differential TSH cutoffs in a 2-screen program. J Clin Endocrinol Metab 2021 106 e338–e349. (10.1210/clinem/dgaa789) 33124651

[20] van Trotsenburg P, Stoupa A, Léger J, et al. Congenital hypothyroidism: a 2020–2021 consensus guidelines update-An ENDO-European Reference Network initiative endorsed by the European Society for Pediatric Endocrinology and the European Society for Endocrinology. Thyroid 2021 31 387–419. (10.1089/thy.2020.0333) 33272083 PMC8001676

[21] Lauffer P, Zwaveling-Soonawala N, Naafs JC, et al. Diagnosis and management of central congenital hypothyroidism. Front Endocrinol 2021 12 686317. (10.3389/fendo.2021.686317) PMC8458656 34566885

[22] Léger J. [Neonatal screening for congenital hypothyroidism]. Med Sci 2021 37 474–481. (10.1051/medsci/2021058) 34003093

[23] Leger J. Screening for congenital hypothyroidism in France. Misdiagnosed cases: collaborative study of screening centres in France. Eur J Pediatr 1990 149 605–607. (10.1007/bf02034742) 2373106

